# Older People’s Needs and Opportunities for Assistive Technologies

**DOI:** 10.1007/978-3-030-51517-1_37

**Published:** 2020-05-31

**Authors:** Jeffrey Soar, Lei Yu, Latif Al-Hakim

**Affiliations:** 8 Digital Research Centre of Sfax, Sfax, Tunisia; 9 Institut Mines-Télécom, CNRS, Paris, France; 10 Université de Sherbrooke, Sherbrooke, QC Canada; 11 Digital Research Centre of Sfax, Sfax, Tunisia; 12 University of Sfax, Sfax, Tunisia; University of Southern Queensland, Toowoomba, QLD 4350 Australia

**Keywords:** Older people, Needs, Assistive technologies, Systematic review

## Abstract

Older adults experience a disconnect between their needs and adoption of technologies that have potential to assist and to support more independent living. This paper reviewed research that links people’s needs with opportunities for assistive technologies. It searched 13 databases identifying 923 papers with 34 papers finally included for detailed analysis. The research papers identified needs in the fields of health, leisure, living, safety, communication, family relationship and social involvement. Amongst these, support for activities of daily living category was of most interest. In specific sub-categories, the next most reported need was assistive technology to support walking and mobility followed by smart cooking/kitchen technology and assistive technology for social contacts with family member/other people. The research aimed to inform a program of research into improving the adoption of technologies where they can ameliorate identified needs of older people.

## Introduction

Global life expectancy rose from 64.2 years in 1990 to 72.6 years in 2019 [1]. There is increasing interest in and availability of support for people choosing to remain in their own homes and delay or avoid moving to institutional care, with an increasing need to improve access to services at home in health management, rehabilitation nursing and entertainment [2]. This research aimed to identify the state of matching needs with technologies focusing on support in the home environment to support independence in everyday activities. Important technology features include ease of use, security, safety, reliability and use independency as important factors in adoption of assistive technology [3]. There is a need for greater awareness of what smart home and assistive technologies are needed to guide technology developers as well as to increase the understanding of potential users of what is available and how it might benefit them.

“Assistive technology” is an umbrella term referring to a range of specialized technology used by people to support activities of daily living and specific tasks [4]. It is about the use of an array of electronic devices incorporated into everyday objects in order to monitoring the user’s status and provide assistance as needed, including feedback, guidance, alerts or warnings [5]. Assistive technology has evolved with and emerged from information technology, passing from detecting and reporting problems, to assisting with prevention of ill-health and adverse events [6]. Smart home technologies refer to technology for clinical and wellness monitoring of people in their homes and/or promotes independence and quality of life [7]. The smart home and assistive technologies mentioned in this literature review covers use in both indoors and outdoors.

This paper aims to address three issues. First, to review the needs of older people for assistive technologies and smart home technologies by identifying relevant research. The methods involved searching bibliographic databases, to screen according to inclusion and exclusion criteria. Second, the paper aims to map needs with available smart home and assistive technologies according to the findings from identified papers. Third, to identify the knowledge gap of needs from older people and the gap of awareness of technologies available.

## Method - Search Strategy and Eligibility of Study Selection

A search was undertaken of 13 bibliographic databases which included: A). Academic Search Ultimate; B). AHFS Consumer Medication Information; C). Anthropology Plus; D). Applied Science & Technology Source Ultimate; E). Business Source Ultimate; F). CINAHL with Full Text; G). Health Business Elite; H). Health Source - Consumer Edition; I). Health Source: Nursing/Academic Edition; J). Humanities Source Ultimate; K). Mental Measurements Yearbook with Tests in Print; L). Psychology and Behavioral Sciences Collection; and M). Sociology Source Ultimate.

Key words for the search were “Older people”, “Elderly”, “Old aged people”, “Assistive technologies” and “Smart Home Technologies”. There were some synonyms because there is a range of terms that authors may use as keywords. No doubt there will be other relevant research into this topic that has escaped our search which is a limitation of this review.

Before the study, we formulated the eligibility criteria which included: A). the result must focus on older people, while other groups can be involved such as younger people with disabilities, but the result towards other groups must be separately demonstrated in the conclusion of the research; B). The research should be based on empirical evidence, observed and calculated from data, questionnaire or interview, not be discussion papers without a data collection; C). The research should discuss older people’s needs that are significantly beneficial to quality of life, including independent living skills, satisfaction of living, mental status, social involvement, selection of aged care mode, relationship with relatives, etc.; D). The factors discussed in the research positively link to and enhance with greater opportunities of assistive technologies which help older people with quality of life but not in other fields; E). The result should be published within 5 years; F); and the paper should be published in English. According to the assistive technologies mentioned and related to older people’s quality of life, to researcher’s introduction, to what researchers observed in sociology experiment, we classified older people’s needs. The following result showed older people’s needs in each type.

## Results

By searching in 13 bibliographic databases we yielded 923 results. We excluded 386 papers due to duplication leaving 537 studies for screening. Based on the exclusion and inclusion criteria, we identified 34 papers for detailed analysis.

Though different researchers classified older people’s needs in various ways, this paper, which looks into their broad types of needs, required a comprehensive way of classification. Our classifications were informed by Lee and Lim, who divided older people’s need into health, leisure, living/safety and family relationship [8]. They included both indoor and outdoor activities, both physical and mental health, both independent living and interaction with others, both self-well-being conditions and objective environment improvement. We found this approach useful to distinguish different kinds of needs towards technologies, it made fewer overlaps and mixes when mapping to older people’s needs. Based on their method, we refined categories, as a result, this review classified older people’s needs towards smart home and assistive technologies into health, leisure, living, safety, communication, family relationship and social involvement – 6 categories in total. The clear summary of categories, sub-categories with frequencies and identified papers is shown in Table 1.

**Table 1.** The frequency of users’ needs towards technologies

Category	Sub-category	References for articles mentioned	Frequency
Health	Sight/Vision Assistance Technology	[7, 9, 18, 21, 22, 26–28, 37]	9
	Long-term Pain Management	[12, 35]	2
	Rehabilitation Management	[16]	1
	Mood Recording/Management Technology	[6, 19, 24, 27, 30]	5
	Medication Reminder/Treatment	[6, 11, 17–19, 22, 27, 28]	8
	General Health Monitoring Technology	[6, 9, 21, 28, 31]	5
	Cognitive Ability Assistance Technology	[21, 26–28, 30]	5
	Nurse Call System	[6]	1
Leisure	General Recreational/Entertainment Technology	[7, 32]	2
	Tailored Games	[6, 7, 10, 16, 18–20, 34]	8
	Sports Assistive Technology	[7, 25, 28]	3
	Musical Instrument Playing Assistance	[6, 7, 16, 19, 34]	5
	Television and radio	[6, 9, 37]	3
	Travel Assistance	[30]	1
	Education Technology	[6, 32]	2
Living	Automatic Control Technology for Home Appliance	[6, 9, 11, 15, 17, 18, 22, 28, 31, 38]	10
	Gardening/Farming Assistance	[7, 27]	2
	Smart Cooking/Kitchen Technology	[7, 11, 13, 16–18, 21, 22, 24, 27–29]	12
	Toilet Use Assistance	[13, 17, 21, 22, 24, 27–29, 31]	9
	Cleaning and Laundry Assistance	[11, 17, 18, 21, 22, 24, 27–29, 35]	10
	Reaching and Grasping Technology	[9, 15, 16, 18, 21, 22, 24, 27, 29, 38]	10
	Showering Assistance	[13, 16, 18, 21, 22, 24, 27–29, 38]	10
	Dressing Assistance	[17, 18, 21, 22, 24, 27–29]	8
	Walking and Mobility Assistance	[9, 16–22, 24–27, 29, 31, 35, 38]	16
	Eating Reminder and Assistance	[17, 21, 24, 27, 28]	5
	Item Locating System	[11, 17, 28]	3
Safety	Overall Sense of Safety	[3, 6, 25, 32, 33]	5
	Falling Prevention	[17, 18, 23, 28, 31, 35, 38]	7
	Reminder for Declined Memory	[11, 15, 17, 22, 26, 34]	6
	Home/Location Finding Technology	[17, 21, 23, 28, 34, 35]	6
	Technology of Emergency Response/Warning about Potential Hazards	[13, 22–24, 30, 31, 34, 35]	8
	Gas Leakage Detector	[6, 13]	2
	Transportation Assistance	[9, 21, 22]	3
Family Relationship and Social Involvement	Finance Managing Assistance	[7, 17]	2
	Appointment/Issue Reminding Technology	[9, 17, 18, 28, 34]	5
	Shopping Assistance/Delivery	[17, 18, 22, 28, 30]	5
	Video Call System	[6]	1
	Assistance of Social Contacts with Family Member/Other People	[6, 10, 14, 17, 21, 26, 27, 29, 30, 32, 36, 37]	12
	Relative Recognizing Technology	[17]	1
Communication	Personal Communication Technology	[7, 9, 14, 18, 30, 34, 36]	7
	Smart Phone and Computer	[7, 9, 11, 23, 26, 34]	6
	Companionship Technology/Robots	[8, 14, 16, 18, 19, 36, 38]	7
Total			238

Among the 6 categories of needs of older people related to smart home and assistive technologies, “Living” category was the highest priority, which represented 40% of the total concerns, followed by “Safety” (16%), “Health” (15%), “Family Relationship and Social Involvement” (11%), “Leisure” (10%) and “Communication” (8%). Looking into subcategories of specific needs, walking and mobility assistance was the most needed, which was mentioned 16 times by identified researchers, represented 6.7% of the entire spectrum of older people’s needs, followed by social contacts with family member/other people and smart cooking/kitchen technology, which both were mentioned 12 times by identified researchers, represented 5.1% of the entire spectrum of older people’s needs.

Relevant systematic reviews in the last 5 years used the keywords “Elderly”, “Older people”, “Smart Home Technologies” and “Assistive Technologies”, our search found 26 relevant systematic reviews published. These were about older people’s attitude to [9] or adoption of [10] technologies, as well as technology for specific disease [11–14], for social [15] and communication [16], for nursing or caregivers [17], for monitoring [18–20] and mental well-beings [21] - none of them were comprehensively about the whole spectrum of assistive technologies, at the same time, none of them comprehensively based on older people’s broad spectrum of needs. here is a need for a review based on older people’s needs that might be addressed by smart home and assistive technologies.

## Discussion

There are three potential ways to link older people with assistive technologies or smart home technologies. The first one is to develop or innovate technologies as the initial activity and then promote the technology to older people and finally evaluate the result of impact. However, technologies that are acquired in ways that are not congruent with seniors’ personal needs and circumstances run a higher risk of proving to be ineffective or inappropriate resulting in poor levels of adoption [22]. The second way is to focus on older people’s attitude and adoption upon assistive technologies - to optimize user acceptance towards products by identifying and eliminating the barriers of adoption. This includes research that looked at user attitude and acceptance and examined social factors which appropriately supports the relationship between users and service providers [23]. The third way is to listen to older people’s needs and develop, optimize the technologies in specific orientation. Because some older adults experience a misfit between technology and needs, they must see the value of a device to use it [24]. The research reported on in this paper follows the third way, which looks into older people’s detailed and specific needs at the beginning. the paper reviews the existed smart home and assistive technologies that cope with the needs, moreover, the direction of technologies’ innovation.

To investigate older people’s needs, much of the extant research reports on projects that chose the direct way, either by observing the phenomena or by analyzing data and transcript: 11 research reports tested the needs by enrolling older people into a clinical trial, project and intervention/control group to be observed and tested for the performance in real scenario; 20 research reports acquired the answer by questionnaire, survey, face-to-face or telephone interview, and derive the information from the data.

The existing literature reports on research that identified older people’s needs of sight/vision assistance technology, long-term pain and rehabilitation management, mood recording/management technology, medication reminder/treatment, nurse call system, general health monitoring and cognitive ability assistance technology. We found the focus was mostly on the need for sight/vision assistance technology (represented 25% in this category), medication reminder/treatment (represented 22% in this category) and general health monitoring technology (represented 14% in this category). At this point, the highly recommended technologies were low vision assistive devices [25], health monitoring robots [26] and e-readers [27].

As for the needs for leisure, research results indicated that older people had the need for general recreational/entertainment technology, tailored games, sports assistive technology, musical instrument playing assistance, television and radio, travel assistance and education technology. We found that tailored games attracted 33% of research focus, which was the most needed by older people in this category. It was followed by 21% research results seeking for the technology for playing musical instrument. Game system, movie/music player [8], and entertainment console [28] were the most preferred.

The very significant category, living, represented of almost half of older people’s needs towards smart home and assistive technologies. To be specific, walking and mobility assistance were the most focused (represented 25% in this category), followed by smart cooking/kitchen technology (represented 13% in this category). Older people had a rather broad range of needs in everyday living, including automatic control technology for home appliance, gardening/farming assistance, smart cooking/kitchen technology, toilet use assistance, cleaning and laundry assistance, reaching and grasping technology, shower assistance, dressing assistance, walking and mobility assistance, eating reminder and assistance and item locating system. Researchers found physical activity stimulation, home automation [27], smart power outlet, universal remote control [29] to be appropriate for older people.

Safety is a critical aspect for older people’s both indoor and outdoor activities. according to identified papers, older people were concerned about overall sense of safety, falling prevention, reminder for declined memory, home/location finding technology, technology of emergency response/warning about potential hazards, gas leakage detector and transportation assistance. There was no doubt that technology of emergency response/warning about potential hazards was the most focused one (represented 22% in this category), followed by falling prevention (represented 19% in this category). Alarm system [30] was the most significant technology, together with gas/smoke sensor [29] and emergency call devices [31].

Communication, family relationship and social involvement played an important role in older people’s mental health. Nine types of needs were identified, including finance managing assistance, appointment/issue reminding technology, shopping assistance/delivery, video call system, assistance of social contacts with family member/other people, relative recognizing technology, personal communication technology, companionship technology/robots, smart phone and computer. Among them, assistance of social contacts with family member and companionship technology/robots were pointed out by 45% of the researchers concentrating on this field. Video call system and social robots [32, 33] were the most recommended technologies.

Researchers looked into older people’s target [25] and expectations [30, 34, 35] towards assistive technology, or just set the feature of a specific type assistive technologies [36] but did not include comprehensive view of assistive technologies. Some of the previous research focused on motivations [37, 38], barriers [39] and effectiveness [26] of smart home and assistive technologies – they focused more on adoption [8, 40, 41] than needs. Looking at the range of assistive technologies mentioned in the research, some research was broad enough but not specified, which just mentioned the whole range of assistive technology [42–45] or technology used in a very broad field [24, 46–50]. This is not useful enough to guide technology developers to map their detailed products to older people. On the other hand, some research provided very narrow view of assistive technologies [3, 28, 32, 33, 51–53], with only one or two specific technologies introduced.

There appears to be a need for an effective way to analyze and predict older people’s needs that can be matched with the assistive technologies that are available.

## Conclusion

There is existing literature into older people’s needs in the field of health, leisure, living, safety, communication, family relationship and social involvement. Among them, living category was of most interest. To be more specific, assistive technology for walking and mobility were of the most interest by researchers. The information was gained mostly by interview, telephone talk, home visit or observation in a project. Though these methods were direct, liable, accurate, they were less efficient by directly interacting with older people, who might not be able to express their needs well because of inadequate awareness of technology or chronic disease that hinders the ability of communication. Another way to link older people’s needs with technologies was to apply a technology push to older people and check the effectiveness and adoption, which may then cause misfit between older adults’ needs and available technology. A better way may be needed to explore the opportunities for smart homes and assistive technologies neither by directly interviewing older people nor by technology push. One suggestion is that researchers can look into databases related to older people’s health and quality of life – by analyzing the significant associating factors related to older people’s independent living, smart home and assistive technologies contributing these factors, which can be referred as the future needed ones. The other solution might be seeking older people’s needs in aged care service provision. To sum up, better method of exploring older people’s needs and market demand of assistive technologies are required, broader types of older people’s needs are to be discovered, at the same time, more types of assistive technologies are to be suggested by further research.
